# Deciphering genetic diversity in conserved cattle bulls to achieve sustainable development goals

**DOI:** 10.1038/s41598-024-61542-0

**Published:** 2024-05-11

**Authors:** Amod Kumar, Rajeev Anand Kumar Aggarwal, M. S. Tantia

**Affiliations:** 1 Animal Genetics Division, ICAR-National Bureau of Animal Genetic Resource, Karnal, Haryana 132001 India; 2 Animal Genetic Resources Division, ICAR-National Bureau of Animal Genetic Resource, Karnal, Haryana 132001 India

**Keywords:** Cattle bulls, Microsatellite, Genetic diversity, Conservation, Agricultural genetics, Evolutionary biology

## Abstract

The primary objective of Sustainable Development Goal target 2.5 established by the United Nations is to ensure the preservation of genetic diversity in domesticated animals. The ICAR-National Bureau of Animal Genetic Resources in India has been actively engaged in the conservation of cattle and buffalo bull semen for long-term storage. This present study aimed to assess the genetic diversity present in the conserved cattle bull semen, which would aid in determining the most suitable strategy for future conservation management. A total of 192 bull semen belonging to 19 cattle breeds were selected to evaluate genetic diversity using 17 pairs of FAO recommended microsatellite primers. Total 267 alleles were detected across all the samples which indicates substantial amount of allelic variation is being maintained in conserved bulls. Further, all cattle bulls semen conserved showed higher observed heterozygosity than expected heterozygosity which indicates excess genetic diversity in all the populations. The F_ST_, F _IT_ and F_IS_ value across the loci and population is 0.146 ± 0.009, 0.054 ± 0.038, and − 0.105 ± 0.035, respectively, which suggests lack of inbreeding in conserved cattle bull semen. This study has established genetic diversity in conserved cattle semen samples to achieve sustainable development goals. In addition, it provides compelling evidence that the current approach for conserving cattle bull semen is heading in the correct direction.

## Introduction

The importance of animal genetic resources conservation has been realized many decades ago, and it has been on priority at international level^1^. Animal breeds have evolved with combinations of mutation, genetic drift, differential selection pressure by local environment and endemic diseases, available feed and fodder and man-made selection^2^. Thus, they are comprised of unique genetic architecture, and therefore, need to be conserved. India possesses a rich abundance of livestock genetic resources, harboring distinct traits such as disease tolerance, adaptability to the local environment, and sustainability in low-input systems. Presently, there are 212 registered breeds of livestock and poultry in India including 50 breeds of cattle. Recent breed wise livestock census has highlighted existence of some cattle breeds which required immediate intervention for future conservation. Recently, the breed watch list published by ICAR-NBAGR has included cattle breeds like Belahi, Krishna Valley, Pulikulum and Khariar, that urgently need intervention due to their endangered status. Further, socio-economic changes, low milk production level of Indian cattle breeds and introduction of exotic and crossbred germplasm in the native tract of these breeds has increased their vulnerability to genetic erosion.

Conserving the genetic diversity of all the existing breeds is essential for harnessing hybrid vigour, preserving specific genes and gene combinations, and insurance against climate and social changes. However, conservation without enough genetic diversity would not be advisable, as low genetic diversity would lead to inbreeding depression. Furthermore, a broader genetic base is crucial for adapting to future climate change and sustainable animal production system. The National Gene Bank at ICAR—National Bureau of Animal Genetic Resources (NBAGR), Karnal, India has been involved to scientific management and conservation of the indigenous livestock biodiversity. It is imperative that the preserved germplasm is representative of the extant biodiversity to be of any value for conservation and future utilization. Recently, Dutch cattle breeds, conserved at gene bank were genetically characterized to evaluate gene diversity and prioritization for gene banking^3^. Similarly, genetic diversity within and between live poultry lines at Norwegian gene bank were evaluated for estimation of conservation values in terms of providing accurate genetic diversity^4^. The assessment of genetic diversity in major livestock species has been facilitated by the development of panels of microsatellite markers by the Food and Agriculture Organization (FAO) and the International Society for Animal Genetics—FAO Advisory Group (FAO, 2011). Microsatellite markers, which are dispersed throughout the entire genome, can be utilized to evaluate genetic variability. These markers possess polymorphic characteristics, exhibit codominance, and are neutral in terms of selection^5^. Microsatellite panels have been used for generating genetic information for African^6^, Asian^7^ European^8^, and mid-South American cattle breeds^9^. In India, a number of researchers have used these markers for genetic characterization of livestock species, such as cattle, buffalo, sheep, goat, camel and donkey^10–13^. These studies have played a crucial role in exploring genetic diversity and facilitating the establishment of new breeds.

The importance of genetic diversity cannot be overstated, as it plays a vital role in animal health, productivity, adaptation to evolving environments, food security, and conservation efforts. Consequently, it is imperative to acknowledge and safeguard this diversity to secure a sustainable future for both animals and humans. Therefore, the present investigation was aimed to decipher genetic diversity in the conserved semen samples of cattle breeds for deciding future strategy for semen conservation.

## Materials and methods

### Sample collection and DNA extraction

A total of 192 cattle bull semen samples, conserved at the National Gene Bank, ICAR-National Bureau of Animal Genetic Resources, Karnal, were considered for the present study. The whole experimental/research work has been approved by competent authority of the institute. Further, all the semen samples have been used with due permission of Director, ICAR-NBAGR, Karnal. Presently, we are conserving 10–20 animals per breed. These animals were required to show the true characteristics of their breed. In addition, samples were normally collected from the organised government livestock farms in their native geographical tract. These conserved bull semen belong to 19 registered cattle such as Amritmahal (2), Bargur (14), Dangi (10), Gangatiri (2), Gir (9), Haryana (23), Kangayam (9), Kankrej (29), Khillar (14), Krishna Valley (8), Nagori (9), Ongole (9), Ponwar (4), Punganur (10), Rathi (6), Red Kandhari (2), Sahiwal (24), Tharparkar (5), and Vechur (3) breeds of India. Genomic DNA (gDNA) was isolated from semen samples using Phenol–chloroform method as described elsewhere^14^. Subsequently, quality and quantity of extracted gDNA was assessed using a NanoDrop spectrophotometer.

### Microsatellite loci amplification

The isolated genomic DNA (gDNA) was subjected to amplification using the polymerase chain reaction (PCR) technique, employing a selected panel of 17 microsatellite primer pairs recommended by FAO (Table 1). These microsatellite primer pairs were chosen based on their previously demonstrated high polymorphism in Indian cattle populations Sharma et al.^10^. The forward primer of each pair was labelled with fluorochrome dye (FAM, NED, PET and VIC), and manufactured by Applied Biosystems. For amplification of microsatellite loci, ~ 50 ng of gDNA was added to cocktail of 10 pMol of primer pairs and 7.5 ul of 2 × master mix (Thermoscientific) in final volume of 15 ul. All the loci were amplified using Applied Biosystems thermal cycler with the following conditions: initial denaturation of 1 min at 94 °C, 32 cycles of 1 min at 94 °C, 1 min at annealing temperature of each primer, 1 min at 72 °C, and a final extension of 10 min at 72 °C. Further, amplified PCR products were checked on 1 percent agarose gel electrophoresis.

**Table 1:** Details of 17 ISAG/FAO recommended microsatellite primer pairs used for characterization of conserved cattle bulls.

Primers	Primer sequences (5′-3′)	Forward label	Annealing temperature	Product size (base pair)	No. of alleles
BM1824	F-gagcaaggtgtttttccaatc	VIC	58 °C	176–196	9
	R-cattctccaactgcttccttg				
CSSM33	F-cactgtgaatgcatgtgtgtgagc	NED	58 °C	144–188	23
	R-cccatgataagagtgcagatgact				
CSSM66	F-acacaaatcctttctgccagctga	FAM	60 °C	167–207	13
	R-aatttaatgcactgaggagcttgg				
ETH10	F-gttcaggactggccctgctaaca	NED	58 °C	185–221	16
	R-cctccagcccactttctcttctc				
ETH3	F-gatcaccttgccactatttcct	NED	57 °C	90–124	21
	R-acatgacagccagctgctact				
HEL09	F-cccattcagtcttcagaggt	FAM	59 °C	140–182	21
	R-cacatccatgttctcaccac				
ILSTS06	F-tgtctgtatttctgctgtgg	FAM	58 °C	275–303	13
	R-acacggaagcgatctaaacg				
ILSTS34	F-aagggtctaagtccactggc	VIC	59 °C	138–212	26
	R-gacctggtttagcagagagc				
INRA05	F-caatctgcatgaagtataaatat	FAM	54 °C	130–148	9
	R-cttcaggcataccctacacc				
INRA63	F-atttgcacaagctaaatctaacc	PET	54 °C	162–190	8
	R-aaaccacagaaatgcttggaag				
MM12	F-caagacaggtgtttcaatct	PET	52 °C	88–134	18
	R-atcgactctggggatgatgt				
MM8	F-cccaaggacagaaaagact	NED	55 °C	114–144	16
	R-ctcaagataagaccacacc				
TGLA122	F-ccctcctccaggtaaatcagc	VIC	58 °C	133–179	18
	R-aatcacatggcaaataagtacatac				
TGLA227	F-cgaattccaaatctgttaatttgct	PET	55 °C	67–119	12
	R-acagacagaaactcaatgaaagca				
TGLA53	F-gctttcagaaatagtttgcattca	FAM	58 °C	142–184	22
	R-atcttcacatgatattacagcaga				
TGLA122	F-ccctcctccaggtaaatcagc	VIC	58 °C	133–179	18
	R-aatcacatggcaaataagtacatac				
ILSTS11	F-gcttgctacatggaaagtgc	NED	58 °C	249–273	9
	R-ctaaaatgcagagccctacc				

### Microsatellite genotyping

A ready-to-run plate was prepared by mixing 1 μL PCR product, 8.6 μL of Hi-Di formamide and 0.3 μL of LIZ 500 size standard. Subsequently, amplified labelled DNA fragments were electrophoresed on Applied Biosystems Genetic Analyzer. GeneMapper 3.0 software (Applied Biosystems) was employed to extract allele size from. fsa files. Firstly, panels and bins were created for all 17 microsatellite primer pairs. Then, fragment analysis samples files were imported and allele size were extracted from the. fsa files. Further, generated excel files with allele size was used for evaluation of genetic diversity. The stutter related scoring error, often seen in dinucleotide repeats, was absent and alleles could be scored unambiguously.

### Microsatellite statistical analysis

The population genetic descriptive statistics for each breed and microsatellite loci were estimated using GenAlEx v6.5 software^15^. These parameters include: allele frequency, observed number of alleles (No), number of effective alleles (Ne), information index (I), observed heterozygosity (Ho), expected heterozygosity (He), unbiased expected heterozygosity (uHe), and fixation index (F). To investigate the distribution of genetic variability among different breeds, the analysis focused on examining the Wright's F-statistics (specifically F_IS_, F_ST_, and F_IT_. A pairwise matrix of the genetic distances was then utilized to construct heatmap, which was subsequently visualized in excel.

## Results

### Microsatellite polymorphism

Genetic diversity of indigenous cattle bulls conserved at National Gene Bank, ICAR-National Bureau of Animal Genetic Resources, Karnal were established using microsatellite markers. All 17 microsatellite primer pairs used for this study were amplified and confirmed by gel-electrophoresis. The genotype data obtained in the current study revealed that a substantial level of genetic variation is conserved within cattle populations. All the selected microsatellite loci were found 100 percent polymorphic in all the cattle populations except Red Kandhari (94.12%), Gangatiri (94.12%), Amritmahal (82.35%) and Tharparkar (94.12%) cattle breeds. A total number of 267 alleles were identified in the present study. Out of these alleles, ILSTS34 marker contributed highest number of alleles (26) while INRA63 marker contributed least (8).

The number of alleles observed at each locus serves as an indicator of genetic variability, which directly influences the differentiation of breeds within a species. Therefore, the FAO has established a minimum requirement of four alleles per locus for assessing genetic distinctions between breeds. In accordance with this criterion, all 17 microsatellite loci displayed abundant polymorphism, allowing for the evaluation of genetic variability within breeds and the exploration of genetic differences between breeds, as each locus exhibited four or more alleles. The highest number of observed alleles were detected in Haryana bulls (8.647 ± 0.790) while lowest number were detected in Amritmahal (2.235 ± 0.202) with average number of alleles across all the populations and loci of 5.276 ± 0.145 (Table 2). The average number of effective alleles varied from 2.07 ± 0.19 in Amritmahal breed to 4.37 ± 0.49 in Haryana breed with mean average effective alleles across populations of 3.45 ± 0.08. The allelic diversity is the easiest ways to measure genetic diversity as it quantify the number of alleles present in the population^16^. Among all the population studied, highest observed heterozygosity was found in Red Kandhari breed (0.85 ± 0.07) while lowest in Amritmahal breed (0.62 ± 0.10). Further, average observed heterozygosity across the whole population was found to be 0.72 ± 0.01 (Table 2). The expected heterozygosity was found either approximately equal or less than observed heterozygosity in all the populations studied.

**Table 2:** Genetic diversity indices (Average) across 19 conserved cattle breeds with 17 microsatellite markers.

Population	Na*	Ne*	I*	Ho*	He*	uHe*	F*
Tharparkar	4.53 ± 0.49	3.63 ± 0.47	1.26 ± 0.13	0.77 ± 0.07	0.63 ± 0.05	0.71 ± 0.06	0.23 ± 0.05
Nagori	4.76 ± 0.34	3.41 ± 0.32	1.29 ± 0.09	0.68 ± 0.06	0.65 ± 0.04	0.72 ± 0.04	0.05 ± 0.07
Rathi	4.47 ± 0.41	3.22 ± 0.36	1.22 ± 0.11	0.64 ± 0.07	0.62 ± 0.04	0.69 ± 0.05	0.01 ± 0.07
Sahiwal	8.00 ± 0.93	4.09 ± 0.48	1.53 ± 0.13	0.69 ± 0.05	0.68 ± 0.04	0.70 ± 0.04	0.00 ± 0.03
Haryana	8.65 ± 0.79	4.37 ± 0.50	1.62 ± 0.12	0.76 ± 0.05	0.71 ± 0.04	0.73 ± 0.04	0.06 ± 0.03
Gangatiri	2.76 ± 0.25	2.61 ± 0.27	0.90 ± 0.10	0.74 ± 0.09	0.54 ± 0.05	0.72 ± 0.07	0.37 ± 0.10
Ponwar	4.12 ± 0.28	3.22 ± 0.29	1.23 ± 0.08	0.75 ± 0.06	0.65 ± 0.03	0.74 ± 0.03	0.14 ± 0.07
Kankrej	8.29 ± 0.71	3.73 ± 0.38	1.50 ± 0.11	0.68 ± 0.05	0.68 ± 0.04	0.69 ± 0.04	0.00 ± 0.04
Gir	5.41 ± 0.54	3.31 ± 0.39	1.29 ± 0.12	0.65 ± 0.05	0.62 ± 0.04	0.66 ± 0.05	0.07 ± 0.07
Dangi	6.00 ± 0.56	3.86 ± 0.37	1.45 ± 0.1	0.78 ± 0.05	0.69 ± 0.03	0.73 ± 0.03	0.13 ± 0.06
Khillar	6.18 ± 0.41	3.81 ± 0.39	1.47 ± 0.08	0.70 ± 0.04	0.70 ± 0.03	0.73 ± 0.03	0.00 ± 0.04
Krishna Valley	5.41 ± 0.38	3.74 ± 0.37	1.41 ± 0.09	0.66 ± 0.05	0.69 ± 0.03	0.74 ± 0.03	0.06 ± 0.05
Red Kandhari	3.06 ± 0.22	2.92 ± 0.22	1.04 ± 0.09	0.85 ± 0.07	0.61 ± 0.04	0.81 ± 0.06	0.43 ± 0.10
Amritmahal	2.24 ± 0.20	2.07 ± 0.19	0.69 ± 0.10	0.62 ± 0.10	0.44 ± 0.06	0.59 ± 0.08	0.41 ± 0.14
Punganur	5.88 ± 0.38	3.75 ± 0.3	1.45 ± 0.08	0.77 ± 0.05	0.70 ± 0.03	0.74 ± 0.03	0.11 ± 0.06
Ongole	5.53 ± 0.30	3.33 ± 0.28	1.36 ± 0.07	0.69 ± 0.05	0.67 ± 0.03	0.70 ± 0.03	0.02 ± 0.07
Bargur	6.12 ± 0.48	3.84 ± 0.32	1.47 ± 0.08	0.76 ± 0.04	0.71 ± 0.02	0.74 ± 0.02	0.08 ± 0.05
Kangayam	5.41 ± 0.48	3.7 ± 0.34	1.40 ± 0.09	0.73 ± 0.07	0.69 ± 0.03	0.73 ± 0.03	0.03 ± 0.08
Vechur	3.41 ± 0.26	2.89 ± 0.25	1.08 ± 0.09	0.73 ± 0.07	0.60 ± 0.04	0.73 ± 0.05	0.21 ± 0.09
Average	5.27 ± 0.14	3.44 ± 0.08	1.29 ± 0.02	0.71 ± 0.013	0.64 ± 0.01	0.71 ± 0.01	0.11 ± 0.02

*Na: number of alleles, Ne: effective number of alleles, I: information index, Ho: observed heterozygosity, He: expected heterozygosity, uHe: unbiased expected heterozygosity, F: Fixation index.

### Population differentiation

**Figure 1:**
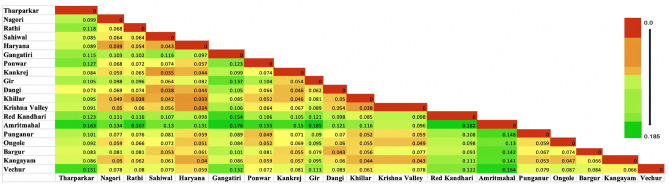
Pairwise Fst estimates between each pair of 19 conserved cattle populations.

The F-statistics results for each of the 17 loci across populations are displayed in Table 3. The overall deficit of heterozygotes across populations (F_IT_) amounted to 5.4%. Notably, the negative value of the overall deficit of heterozygotes (F_IS_) indicated the absence of inbreeding within the analyzed populations. The multi-locus F_ST_ values, which signify breed differentiation, demonstrated that 14.6% of the total genetic variation resulted from distinct allelic differences between the breeds. The remaining 86.6% of genetic variation corresponded to differences among individuals within the breed across the 17 markers. It is important to note that all loci contributed to the observed differentiation, with the highest values observed for ETH3 (19.9%). The pair-wise F_ST_ values of breeds, as illustrated in Fig. 1, ranged from 0.033 to 0.185. This indicates the least differentiation between Khillar-Haryana (0.033), while the greatest divergence was observed between Gir and Amritmahal (0.185).

**Table 3:** Global F-Statistics (Fis: Inbreeding coefficient, Fit: Overall fixation index, Fst: Fixation index, Nm: number of effective migrants) for each of 17 microsatellite loci analyzed across all cattle populations.

Locus	Fis	Fit	Fst	Nm
CSSM33	− 0.158	− 0.029	0.112	1.980
HEL09	− 0.197	− 0.035	0.136	1.588
ILSTS06	0.040	0.221	0.188	1.077
ILSTS34	− 0.085	0.062	0.136	1.592
BM1824	− 0.204	− 0.017	0.156	1.355
CSSM66	− 0.065	0.094	0.150	1.422
ETH3	− 0.240	0.006	0.199	1.007
ILSTS05	− 0.150	− 0.004	0.127	1.723
MM12	− 0.076	0.098	0.161	1.300
ETH10	− 0.102	0.087	0.171	1.210
INRA05	− 0.092	0.029	0.111	2.003
INRA63	0.006	0.150	0.145	1.479
TGLA227	− 0.085	0.060	0.133	1.626
ILSTS11	− 0.525	− 0.422	0.068	3.420
MM8	0.056	0.237	0.192	1.049
TGLA122	0.120	0.292	0.195	1.032
TGLA53	− 0.033	0.080	0.109	2.038
Mean ± SE	0.105 ± 0.035	0.054 ± 0.038	0.146 ± 0.009	1.582 ± 0.141

## Discussion

National Gene Bank, ICAR-NBAGR, Karnal is dedicated to achieve Sustainable Development Goals (SDG) through conserving genetic diversity through preserving semen, somatic cell, and embryos for long term storage. Along with the conservation of animal genetic resources, assessment of genetic diversity is crucial for preserving genetic diversity and preventing the loss of undesirable alleles. This finding of this study revealed excessive heterozygosity across all the cattle populations conserved at National Gene Bank, ICAR-NBAGR. This statement can be validated by comparing the expected (0.65 ± 0.01) and observed (0.72 ± 0.01) heterozygosity across all the cattle population conserved.

In this study, a total of 267 alleles were identifed across all the 192 cattle bulls samples using 17 microsatellite markers (1.36 alleles/individual). However, previous studies revealed 1.22^17^, 1.25^18^, and 0.707^10^ alleles per individual in Indian cattle populations. The detection of a higher number of alleles per individual in conserved cattle bulls indicates that substantial amount of allelic variation is being maintained at National Gene Bank. Moreover, it is worth mentioning that smaller number of microsatellite primer pairs (17) were used for this study as compared to the previous studies, which again provides an indication towards existence of sufficient allelic variation in the conserved semen samples. Interestingly, ILSTS34 marker contributed highest number of alleles (26) in the selected individuals, which is well corroborated with the previous studies^10,18^.

In this study, we observed wide range of average observed number of alleles per locus, ranging from 2.235 ± 0.202 in Amritmahal to 8.647 ± 0.790 in Haryana cattle. This variation may be attributed to significant differences in the sample sizes of the conserved cattle populations at the National Gene Bank. Further, average observed number of alleles across all the populations and loci was 5.276 ± 0.145, and was lower than other research reports published elsewhere^10,18,19^. However, when comparing specific breeds, the allelic diversity in Sahiwal cattle (8.0 ± 0.928) and Haryana cattle (8.647 ± 0.790) was found to be higher than what was previously reported in studies by Mukesh et al.^17^ and Sharma et al.^10^. At global level, less allelic diversity was observed across all the populations as compared to exotic breeds such as Burlina, Brown Swiss and Holstein Friesian cattle^20^. Additionally, lower value of effective number of alleles as compared to observed number of alleles across all the cattle populations suggested that there were many low frequency alleles in the populations. This reduced allelic diversity in the current scenario can be attributed to the smaller sample sizes per breed compared to previous studies. It is recommended that maximum allelic diversity be conserved in various Gene Banks established worldwide to ensure future sustainability.

The detection of a high level of observed heterozygosity (0.72 ± 0.01) across all loci and populations in the conserved cattle bulls signifies a remarkable degree of genetic diversity. This can be attributed to a reduced influence of human-driven selection pressures and suggests the presence of large effective population sizes in the considered Indian cattle populations. The substantial genetic variation observed in Indian cattle breeds has likely contributed to their adaptability across diverse agroclimatic regions. This genetic diversity is likely a result of environmental pressures for adaptability and natural processes of mutation. The indigenous Indian cattle populations, managed according to local use and traditional husbandry practices, have shown no signs of inbreeding issues and have successfully maintained a higher level of genetic variability. This enhanced genetic diversity has played a crucial role in their superior adaptation to the natural environment. This genetic diversity can be well exploited for cattle genetic improvement as well as to facilitate rapid adaptation to changed breeding goals^21^. Genetic diversity is essential for any population to adapt and survive in their environments. It also facilitates local population or breed adaptation to dynamic environments. Further, leveraging high genetic diversity becomes crucial for expanding the genetic pool when a concerned breed or population confronts issues such as inbreeding and diminished genetic diversity, which in turn increases the risk of extinction.

The overall estimate of observed heterozygosity in the present investigation (0.72 ± 0.01) was higher than previous investigations such as Tharparkar (0.643) and Rathi (0.694) cattle^22^, Kherigarh cattle^19^, and 15 other Indian cattle breeds^23^. Moreover, it was found higher than Indonesian cattle breeds^24^ Hartón del Valle, Angus, Brangus, Holstein, and Senepol cattle breeds in Colombia Montoya et al.^25^ and selected Ethiopian indigenous cattle^26^. An interesting observation was made in this study, wherein it was found that expected heterozygosity is either equal or less than observed heterozygosity in all the populations under investigation. It is worth mentioning that many studies explaining genetic diversity using microsatellite markers have found out less observed heterozygosity than expected heterozygosity^10,17,20,22,27^ except few^28^. This further confirms that a substantial level of genetic diversity is being effectively maintained in the conserved cattle bulls at the National Gene Bank, ICAR-NBAGR.

All cattle populations conserved at National Gene Bank revealed no heterozygote deficit except the Amritmahal cattle (0.058). These finding may be interpreted as cattle bulls conserved might be produced through outcrossing. Further, these results are well corroborated with the pattern expressed in estimates of heterozygosity and suggests lack of inbreeding in the conserved cattle bulls The National Gene Bank's long-term efforts in conserving cattle bulls have successfully preserved high levels of genetic diversity. In India, lack of structured breeding programme at the village level and not culling of cattle bulls may contribute to the maintenance of substantial genetic diversity within and between Indian cattle populations. In contrast, many Indian cattle populations have revealed significant homozygote excess in the previous study^10,22^. This heterozygote deficit might be due to collection of samples from closed herd or from sampling error.

Wright’s F-statistics, and particularly F_ST_, are valuable tools for understanding the evolutionary processes that shape the structure of genetic variation within and between populations, and they are among the most widely used descriptive statistics in population and evolutionary genetics. In population differentiation, a F_ST_ value greater than 0.15 is typically considered significant^29^. The highest F_ST_ value were found between Gir and Amritmahal cattle (0.185), Red Kandhari and Amritmahal (0.182), and Gangatiri and Amritmahal (0.176). This result revealed within-breed genetic variation is more than between-breed genetic variation. Further, this genetic variation could be well utilised for genetic upgradation and conservation of cattle populations in India. Further, the overall F_ST_, F _IT_ and F_IS_ value across the loci and population is 0.146 ± 0.009, 0.054 ± 0.038, and − 0.105 ± 0.035, respectively. These estimates obtained in the present investigation suggests lack of inbreeding in the conserved bull semen. However, these type of findings are rarely observed in natural conditions. Mostly, F_IS_ would be positive and F_IT_ > F_ST_, this could be considered as evidence of inbreeding^30^. It is commonly hypothesized that in a population where mating occurs randomly, genes would exhibit equal levels of relatedness both within individuals and between individuals. In such conditions F_IT_ equals F_ST_ or F_IS_ equals zero^22^. Sodhi et al.^22^ reported *F*-statistics: F_IS_ = 0.112 ± 0.029, F _IT_ = 0.169 ± 0.033, F _ST_ = 0.065 ± 0.017, and interpreted departure of populations from random mating. In addition, across all the loci under investigation, F_ST_ ranged from 0.068 (ILSTS11) to 0.199 (ETH3) with an average of 0.146. This F_ST_ values revealed that the most of total allelic variation (85.4%) corresponds to differences among individuals, and only 14.5% genetic variation could be attributed to differences among breeds. Further, ETH3 (0.199), TGLA122 (0.195), MM8 (0.192), ILSTS06 (0.188), ETH10 (0.171), MM12 (0.161), BM1824 (0.156) markers might be considered as more informative to differentiate the populations under investigation. However, this statement need to be validated in large number of individuals of populations under study.

## Conclusion

The present investigation established the genetic diversity and differentiation among the bulls, conserved at ICAR-NBAGR Karnal and contributed to the attainment of Sustainable Development Goals. The findings highlight that conserved bull semen demonstrates noteworthy heterozygosity, as evidenced by the analysis of 17 FAO-recommended microsatellite primer pairs. Further, large number of alleles were identified across all samples, indicating a significant level of allelic variation is being preserved in conserved cattle bulls. Importantly, no signs of inbreeding were detected within any of the populations examined. This study indicates that the approach for selecting cattle bulls for semen conservation is heading in the correct direction.

## Data Availability

All relevant data has been mentioned within the manuscript.
